# Implementation of the Realized Genomic Relationship Matrix to Open-Pollinated White Spruce Family Testing for Disentangling Additive from Nonadditive Genetic Effects

**DOI:** 10.1534/g3.115.025957

**Published:** 2016-01-19

**Authors:** Omnia Gamal El-Dien, Blaise Ratcliffe, Jaroslav Klápště, Ilga Porth, Charles Chen, Yousry A. El-Kassaby

**Affiliations:** * Department of Forest and Conservation Sciences, Faculty of Forestry, University of British Columbia, Vancouver, V6T 1Z4, Canada; † Pharmacognosy Department, Faculty of Pharmacy, Alexandria University, 21521, Egypt; ‡ Department of Genetics and Physiology of Forest Trees, Faculty of Forestry and Wood Sciences, Czech University of Life Sciences Prague, 165 21 Praha 6, Czech Republic; § Department of Biochemistry and Molecular Biology, Oklahoma State University, Stillwater, Oklahoma 74078

**Keywords:** open-pollinated families, genetic variance decomposition, pedigree- and marker-based relationships, Mendelian sampling term, GenPred, genomic selection, shared data resource

## Abstract

The open-pollinated (OP) family testing combines the simplest known progeny evaluation and quantitative genetics analyses as candidates’ offspring are assumed to represent independent half-sib families. The accuracy of genetic parameter estimates is often questioned as the assumption of “half-sibling” in OP families may often be violated. We compared the pedigree- *vs.* marker-based genetic models by analysing 22-yr height and 30-yr wood density for 214 white spruce [*Picea glauca* (Moench) Voss] OP families represented by 1694 individuals growing on one site in Quebec, Canada. Assuming half-sibling, the pedigree-based model was limited to estimating the additive genetic variances which, in turn, were grossly overestimated as they were confounded by very minor dominance and major additive-by-additive epistatic genetic variances. In contrast, the implemented genomic pairwise realized relationship models allowed the disentanglement of additive from all nonadditive factors through genetic variance decomposition. The marker-based models produced more realistic narrow-sense heritability estimates and, for the first time, allowed estimating the dominance and epistatic genetic variances from OP testing. In addition, the genomic models showed better prediction accuracies compared to pedigree models and were able to predict individual breeding values for new individuals from untested families, which was not possible using the pedigree-based model. Clearly, the use of marker-based relationship approach is effective in estimating the quantitative genetic parameters of complex traits even under simple and shallow pedigree structure.

Open-pollinated (OP) (also known as wind-pollinated) family testing is, by far, the simplest and most economical means for screening, evaluating, and ranking large numbers of candidate parent trees. Thus, OP testing combines the simplest known field experimental design in pedigree testing as candidate trees enter the test as maternal parents and their offspring are assumed to represent independent half-sib families. OP testing has been widely implemented for several tree species throughout the world, *e.g.*, radiata pine (*Pinus radiata* D. Don) (Burdon and Shelbourne 1971), interior spruce [*Picea glauca* (Moench) Voss × *P. engelmannii* Parry ex Engelm.] (Kiss and Yanchuk 1991), Douglas-fir [*Pseudotsuga menziesii* (Mirb.) Franco] (El-Kassaby and Sziklai 1982; Johnson 1997), western larch (*Larix occidentalis* Nutt.) (Ratcliffe *et al.* 2014), and Scots pine (*P. sylvestris* L.) (Korecky *et al.* 2013), and it is often considered as a prelude to full-pedigree testing (Jayawickrama and Carson 2000).

Genealogically speaking, OP testing (*i.e.*, partial pedigree) is positioned between the “no pedigree” provenance testing (Callaham 1964) and the “full-pedigree” mating design-based progeny testing that includes all higher levels of relatedness and connectivity among the created families (Namkoong *et al.* 1988). Thus, the accuracy of all OP testing-based estimated genetic parameters (*e.g.*, additive genetic variance, heritability, breeding values, *etc*.) is superior to the former yet somewhat limited compared to the latter (but also see, Hallingback and Jansson 2013). In fact, doubts are often raised regarding the accuracy of OP family testing-derived genetic parameters as the assumption of “half-sibling” is hardly fulfilled (Namkoong 1966; Squillace 1974; Askew and El-Kassaby 1994).

The pedigree-based genetic relationships among individuals [based on the so-called ***A***-matrix: average numerator relationship matrix (Wright 1922)] are often used to estimate the genetic variance components by using the Restricted Maximum Likelihood (Gilmour *et al.* 1995) and predict each individual’s breeding value using the Best Linear Unbiased Prediction algorithms (Henderson 1975, 1976, 1984). However, while effective, this method with its traditional pedigree-based approach does not adjust for the Mendelian sampling term, that is, this method ignores variation among family members of a half- or full-sib family around the family’s average relatedness [as all sibs are not alike (Hill and Weir 2011)]. Furthermore, the utilization of the ***A***-matrix, specifically, in the case of the well-known “shallow” pedigree present within most forest tree breeding and testing populations does not permit detecting hidden coancestry and inbreeding. Consequently, individuals’ estimated breeding values are inflated by the overestimation of the additive genetic variance.

With the affordability, scalability, and high-throughput nature of next generation sequencing technologies, tens of thousands of single nucleotide polymorphisms (SNPs) have become available for model and nonmodel species (Baird *et al.* 2008; Elshire *et al.* 2011; Peterson *et al.* 2012; Poland *et al.* 2012; Truong *et al.* 2012; Chen *et al.* 2013). This technical advancement made it possible to ascertain, with a great level of accuracy, the actual fraction of alleles shared between individuals, and the estimates of the individuals’ pairwise realized relationship including potential inbreeding can be easily determined (Santure *et al.* 2010). Therefore, genomic fingerprinting data permit the accurate estimation of the realized relationships among any set of individuals, irrespective of their genealogy, to construct the realized genomic relationship matrix (***G***-matrix) which can be used to substitute the ***A***-matrix (VanRaden 2008). This advancement represents a clear quantitative genetics watershed as the complete dependency on known pedigree relationships (*i.e.*, ***A***-matrix) for estimating genetic parameters can be circumvented in the so-called “pedigree-free models” using the ***G***-matrix. As already demonstrated in earlier cases, the ***G***-matrix can provide relatively accurate genetic variance components and breeding value estimates without the need for elaborate mating designs (Thomas *et al.* 2002; Frentiu *et al.* 2008; Hayes *et al.* 2009; El-Kassaby *et al.* 2012; Gay *et al.* 2013; Porth *et al.* 2013; Zapata-Valenzuela *et al.* 2013; Klápště *et al.* 2014; Muñoz *et al.* 2014).

The use of the ***G***-matrix in OP family testing has several implications and is expected to: (1) overcome the drawback of the average numerator relationship matrix (***A***-matrix) as genomic data will unravel any undetectable hidden relatedness such as full-sibs, self-sibs, and self-halfs that inflates the estimated additive genetic variance (Namkoong 1966; Squillace 1974; Askew and El-Kassaby 1994) (Figure 1), (2) provide more accurate genetic covariances among relatives, thus accounting for the Mendelian sampling term (Visscher *et al.* 2006), and (3) provide higher flexibility in capturing the allele frequency segregation in quantitative trait loci (QTL) (present *vs.* absent QTL) (Lippert *et al.* 2013). Additionally, we hypothesize that also for OP families the use of genomic markers will create an opportunity to effectively decompose the genetic variance components, thus separating the additive and nonadditive genetic components through the definition of realized genomic relationship matrix related to specific variance components, a so far unattainable feat for OP family testing.

**Figure 1:**
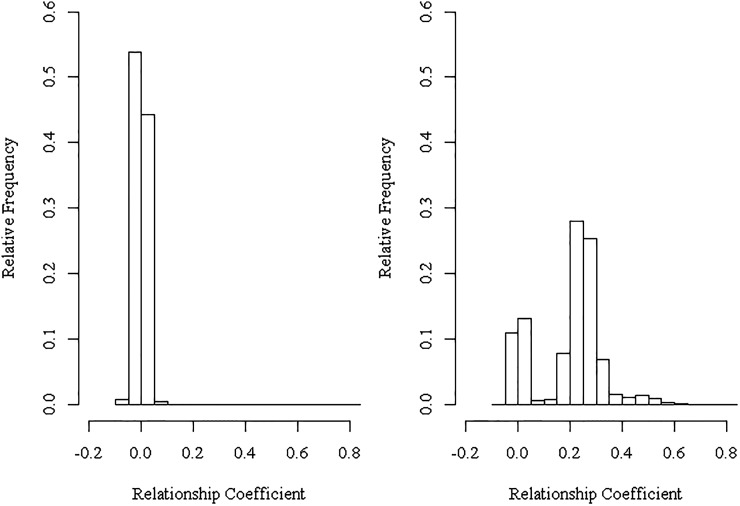
Representative histograms of the genomic pairwise relationship coefficients within (right panel) and among (left panel) members of the 214 white spruce OP families showing relationships clustering around the expected 0.25 with deviations from 0.25 as indicative of imperfect half-sib family (right panel) and clustering around 0.00 as indicative of no relationship (left panel).

Here, we used 214 white spruce OP families grown on one site [Mastigouche Arboretum, Quebec, Canada (Lat. 46° 38’ N, Long. 73° 13’ W, Elev. 230 m)] in a randomized complete block design, replicated over six blocks (replications) with each OP family represented by five-tree row plots within each of the six blocks [for complete details, see Beaulieu *et al.* 2014)].We compared the genetic variance estimates generated from both the average numerator relationship matrix (the expected relationships) and the realized genomic relationship matrix (the observed relationships) to demonstrate the genomic markers’ utility in partitioning the genetic variance components into additive and nonadditive effects. To our knowledge, this study provides the first attempt of such an analysis approach in OP families.

## Materials and Methods

### White spruce open-pollinated progeny test, phenotype data, and genotyping

The white spruce [*P. glauca* (Moench) Voss] phenotypic and genotypic data used are available online from the Dryad Digital Repository: doi:10.5061/dryad.6rd6f (Beaulieu *et al.* 2014). Briefly, the study site is part of a larger, three-site white spruce provenance-progeny test established in 1979 by the Canadian Forest Service in Quebec, Canada. Each site was planted as a randomized complete block design with six blocks and five-tree row plots at 1.2 and 2.4 m spacing within and between rows, respectively. The present study is based on a subset of the provenance-progeny test that includes eight individuals for each OP family from 214 families representing a total of 1694 individuals; the average family representation per block was 1.32 trees since not all families were present in all blocks. It is noteworthy to state that the 214 OP families were selected from 43 provenances throughout Quebec, thus a population effect might be present. Beaulieu *et al.* (2014), using principal component analysis, reported the presence of weak population structure with no defined geographical pattern. In fact, Beaulieu *et al.* (2014) estimated that 1.3% of the total variance was explained by the first two principal component analysis eigenvectors and indicated that their lack of population structure is concordant with previous studies using the same populations, thus population structure was not considered in the present study. Wood density was determined using X-ray densitometry from 12 mm increment cores collected 1.3 m from the ground (see Beaulieu *et al.* 2011, for details). Trees were genotyped for 7338 SNP loci from 2814 genes using Illumina Infinium HD iSelect bead chip PgAS1 (Illumina, San Diego, CA) (for details see Rigault *et al.* 2011). The data used are available from the Dryad Digital Repository: doi: 10.5061/ dryad.6rd6f (Beaulieu *et al.* 2014).

### Relationship matrices

The additive relationship matrix was estimated as follows:

$$G_{\mathrm{add}}=\frac{\mathrm{ZZ}\prime }{2\sum p_{i}(1-p_{i})}$$ [1]

where ***Z*** is the rescaled genotype matrix following ***M*** − ***P***, ***M*** is the genotype matrix containing genotypes coded as 0, 1, and 2 according to the number of alternative alleles, and ***P*** is a vector of twice the allelic frequency, *p* (VanRaden 2008). The dominance genetic variance was fitted by including a marker-based dominance relationship matrix as follows:

$$G_{\mathrm{dom}}=\frac{\mathrm{WW\prime}}{{\left(2pq\right)}^{2}}$$ [2]

where ***W*** is the matrix containing −2*q*^2^ for the alternative homozygote, 2*pq* for the heterozygote, and −2*p*^2^ for the reference allele homozygote (Vitezica *et al.* 2013). Similarly, epistatic variance was fitted by including several relationship matrices capturing first-order additive × additive, dominance × dominance, and additive × dominance interaction. The relationship matrices were constructed as the Hadamard product of the relationship matrices defined above: ***G*_add_**#***G*_add_**, ***G*_dom_**#***G*_dom_**, and ***G*_add_**#***G*_dom_** (Su *et al.* 2012; Muñoz *et al.* 2014).

The variance components from pedigree-based analysis (ABLUP) were obtained by solving the following mixed model:

$$y=\mathrm{X\beta}+Z_{i}u+Z_{j}r+Z_{k}\mathrm{rxf}+e$$ [3]

where ***y*** is the vector of phenotypic measurements, ***β*** is the vector of fixed effects (overall mean), ***u*** is the vector of random additive genetic effects following ***u*** ∼ N(0, ***A$\sigma_{a}^{2}$***), where ***A*** is the average numerator relationship matrix and $\sigma_{a}^{2}$ is the additive genetic variance, ***r*** is the vector of random replication effect following ***r*** ∼ N(0, ***I$\sigma_{r}^{2}$***), where $\sigma_{r}^{2}$ is the replication variance, ^***rxf***^is the vector of random replication × family interaction effects following ^***rxf***^ ∼ N(0, ***I$\sigma_{\mathrm{rxf}}^{2}$***), where $\sigma_{\mathrm{rxf}}^{2}$ is the replication × family interaction variance, and ***e*** is a vector of the random residual effects following ***e*** ∼ N(0, ***I$\sigma_{e}^{2}$***), where $\sigma_{e}^{2}$ is the residual error variance, ***X***, ***Z_i_***, ***Z_j_***, and ***Z_k_*** are incidence matrices relating fixed and random effects to measurements in vector ***y***.

The variance components from the analysis using the marker-based additive relationship matrix (GBLUP-A) were obtained from the model described above but the average numerator relationship matrix ***A*** is substituted by the marker-based relationship matrix ***G_add_***. The extended model for the dominance terms are performed as follows:

$$y=\mathrm{X\beta}+Z_{i}u+Z_{l}d+Z_{j}r+Z_{k}\mathrm{rxf}+e$$ [4]

where ***d*** is the vector of the random dominance effect following ***d*** ∼ N(0, ***G_dom$\sigma_{d}^{2}$_***) where $\sigma_{d}^{2}$ is the dominance variance. Additional model extension for epistatic terms is performed as follows:

$$y=\mathrm{X\beta}+Z_{i}u+Z_{l}d+Z_{m}\mathrm{axa}+Z_{n}\mathrm{dxd}+Z_{p}\mathrm{axd}+Z_{j}r+Z_{k}\mathrm{rxf}+e$$ [5]

where ***axa*** is the vector of random additive × additive epistatic interaction effects following ***axa*** ∼ N(0, ***G_add#add$\sigma_{axa}^{2}$_***), where $\sigma_{axa}^{2}$ is the additive × additive epistatic interaction variance, ***dxd*** is the vector of random dominance × dominance epistatic interaction effects following ***dxd*** ∼ N(0, ***G_dom#dom$\sigma_{dxd}^{2}$_***), where $\sigma_{dxd}^{2}$ is dominance × dominance epistatic interaction variance, and ***axd*** is the vector of random additive × dominance epistatic interaction effects following ***axd*** ∼ N(0, ***G_add#dom$\sigma_{axd}^{2}$_***), where $\sigma_{axd}^{2}$ is the additive × dominance epistatic interaction variance.

Narrow-sense heritability was estimated as ${\hat{h}}^{2}={\hat{\sigma }}_{a}^{2}/{\hat{\sigma }}_{p}^{2}$, where ${\hat{\sigma }}_{a}^{2}$ represents the estimate of the additive variance and ${\hat{\sigma }}_{p}^{2}$ equals the sum of ${\hat{\sigma }}_{e}^{2}\;$ and all random model effect variance component estimates such as additive, dominance, additive × additive, additive × dominance, and dominance × dominance interactions following that of the ABLUP and GBLUPs (GBLUP-A, GBLUP-AD, and GBLUP-ADE) models, respectively (Table 1). The analyses and the derived genetic and environmental parameters and their SEs for the ABLUP and GBLUPs were estimated using ASReml v. 3.0 software (Gilmour *et al.*, 2009).

**Table 1:** Estimates of genetic variance components and their SEs for height (HT) and wood density (WD) for the Québec white spruce population across the four genetic models

		ABLUP			GBLUP-A			GBLUP-AD			GBLUP-ADE			GBLUP-AE	
Trait	Source of Variation	Value (SE)	%		Value (SE)	%		Value (SE)	%		Value (SE)	%		Value (SE)	%
HT	$\sigma_{\mathrm{Rep}}^{2}$	561.4 (383.72)	4.70		554.8 (379.47)	4.68		555.9 (380.27)	4.69		555.3 (379.85)	4.69		555.2 (3.80E+02)	4.69
	$\sigma_{\mathrm{F*Rep}}^{2}$	2624.8 (497.90)	21.97		2653.7 (479.62)	22.38		2658.6 (479.60)	22.43		2614.4 (481.24)	22.08		2613.1 (480.94)	22.07
	$\sigma_{A}^{2}$	2178.9 (879.65)	18.24		1404.0 (413.19)	11.84		1385.3 (413.98)	11.69		1160.9 (482.52)	9.80		1159.0 (480.98)	9.79
	$\sigma_{D}^{2}$	N/A			N/A			133.29 (391.83)	1.13		12.15 (406.64)	0.10		N/A	
	$\sigma_{\mathrm{AA}}^{2}$	N/A			N/A			N/A			1334.8 (1664.2)	**11.27***a*		1352.7 (1595.60)	**11.43**
	$\sigma_{\mathrm{DD}}^{2}$	N/A			N/A			N/A			9.86E-03 (2.23E-03)	0.00		N/A	
	$\sigma_{\mathrm{AD}}^{2}$	N/A			N/A			N/A			9.86E-03 (2.23E-03)	0.00		N/A	
	$\sigma_{E}^{2}$	6581.7 (808.23)	55.09		7243.6 (535.33)	61.10		7119.8 (640.48)	60.06		6163.2 (1391.3)	52.05		6159.1 (1390.90)	52.02
	$h^{2}$	0.249 (0.095)			0.162 (0.046)			0.160 (0.045)			0.134 (0.055)			0.134 (0.054)	
	AIC	17,478.64			17,465.80			17,467.66			17,472.94			17,466.94	
WD*b*	$\sigma_{\mathrm{Rep}}^{2}$	1.36E-05 (1.11E05)	1.07		1.24E-05 (1.04E-05)	1.01		1.26E-05 (1.05E-05)	1.02		1.34E-05 (1.10E-05)	1.10		1.34E-05 (1.10E-05)	1.10
	$\sigma_{\mathrm{F*Rep}}^{2}$	2.47E-05 (4.77E-05)	1.95		5.89E-05 (4.70E-05)	4.78		5.88E-05 (4.69E-05)	4.78		4.65E-05 (4.65E-05)	3.83		4.65E-05 (4.65E-05)	3.83
	$\sigma_{A}^{2}$	7.48E-04 (1.28E-04)	59.01		3.51E-04 (5.52-E05)	28.50		3.48E-04 (5.52E-05)	28.25		2.07E-04 (5.85E-05)	17.05		2.07E-04 (5.85E-05)	17.05
	$\sigma_{D}^{2}$	N/A			N/A			3.50E-05 (4.88E-05)	2.84		7.90E-11 (2.78E-11)	0.00		N/A	
	$\sigma_{\mathrm{AA}}^{2}$	N/A			N/A			N/A			6.32E-04 (1.34E-04)	**52.03**		6.32E-04 (1.34E-04)	**52.03**
	$\sigma_{\mathrm{DD}}^{2}$	N/A			N/A			N/A			5.05E-10 (1.78E-10)	0.00		N/A	
	$\sigma_{\mathrm{AD}}^{2}$	N/A			N/A			N/A			5.05E-10 (1.78E-10)	0.00		N/A	
	$\sigma_{E}^{2}$	4.81E-04 (1.12E-03)	37.96		8.10E-04 (6.28E-05)	69.71		7.77E-04 (7.62E-05)	63.11		3.16E-04 (1.11E-04)	25.98		3.16E-04 (1.11E-04)	25.98
	$h^{2}$	0.609 (0.093)			0.303 (0.043)			0.300 (0.043)			0.179 (0.049)			0.179 (0.049)	
	AIC	−9687.42			−9716.32			−9714.86			−9726.64			−9732.64	

a numbers in bold highlight additive x additive genetic variance

b log transformation.

Models were compared using the Akaike Information Criterion (AIC) estimates obtained from each analysis (Gilmour *et al.* 2009) and the precision of the estimated variance components and their dependence was assessed by investigation of accumulated eigenvalues of the asymptotic sampling correlation matrix of variance component estimates ***F***, where ***F*** = ***L***^-1/2^***VL***^-1/2^ using the asymptotic variance–covariance matrix of estimates of variance components ***V*** and its diagonal matrix ***L*** (Muñoz *et al.* 2014).

A 10-fold cross-validation scenario with five replications was used to assess prediction accuracy and consistency within and between the various models, respectively. Folding of the training population was either random, block restricted, or family restricted. The latter scenario removes the genetic relatedness between the training and validation populations according to the pedigree information. That is, all individuals belonging to a single OP family were strictly assigned to either the training or validation population. Block restricted folding was performed as a leave one block out scenario. That is, all individuals belonging to a single block were assigned as the validation population, while the individuals belonging to the remaining five experimental blocks were randomly divided into 10 folds as the training population. Random folding had no prior restriction when assigning the folds.

Prediction accuracy within and consistency between models was evaluated using the mean Pearson correlation from the five replications. Specifically, the correlation values for each replication were calculated as:

$$r_{EBV_{l},PBV_{mn}}=\frac{cov(EBV_{l},PBV_{mn})}{\sigma_{EBV_{l}}\sigma_{PBV_{mn}}}$$ [6]

where EBV refers to the individual additive breeding value of the validation population obtained using the entire data set (1694 individuals) for the *l*^th^ model (ABLUP, GBLUP-A, GBLUP-AD, GBLUP-ADE), PBV is the individual additive breeding value of the validation population obtained using the *m*^th^ model (ABLUP, GBLUP-A, GBLUP-AD, GBLUP-ADE) and *n*^th^ cross-validation scenario (random, block, family), cov is the covariance, and *σ* is the standard deviation. SEM for the correlations was computed using the following equation:

$$SE=\frac{\sigma }{\sqrt{n}}$$ [7]

where *σ* is the standard deviation of the Pearson correlations and *n* is the number of replicates.

### Data availability

The data used are available from the Dryad Digital Repository: doi: 10.5061/ dryad.6rd6f (Beaulieu *et al.* 2014).

## Results

As expected, replication and family × replication interaction produced constant variance components across the four studied models for both height (4.7 and 22%) and wood density (1 and 2–5%), leaving most of the within-replication effects residing within the residual terms (Table 1). The greatest observed difference between the pedigree-based (ABLUP) and the marker-based (GBLUP-A) models was the substantial discrepancy of the additive genetic variance estimates’ magnitude (Table 1). The additive genetic variances estimated from GBLUP-A were 64.4 and 46.9% of those from the ABLUP for height and wood density, respectively (Table 1). Naturally, this change is reflected in the residual terms as they increased to 110.1 and 168.4% of that of the ABLUP and subsequently resulting in substantial reduction of narrow-sense heritability estimates (${\hat{h}}^{2}$: from 0.25 down to 0.16 for height and from 0.61 down to 0.30 for wood density comparing ABLUP *vs.* GBLUP-A, respectively). Overall, narrow-sense heritability was reduced by 65% for height and 50% for wood density when the genomic relationship matrix GBLUP-A was employed (Figure 1), highlighting known caveats of OP progeny testing. Also, the inflation of additive genetic variance observed in ABLUP and the subsequent impact on heritability estimates were expected, thus it is more reasonable to use the results from the GBLUP-A as the basis for comparing the extended analyses that included dominance (GBLUP-AD), and epistasis and dominance (additive × additive, dominance × dominance, and additive × dominance first-order interaction) (GBLUP-ADE).

The GBLUP-AD analysis produced identical results to that of the GBLUP-A confirming the existence of minuscule and nonsignificant dominance variance estimates, accounting for 1.13 and 2.84% of the total phenotypic variance for height and wood density, respectively (Table 1). This is not surprising considering the small sample size of the studied OP families (≈8 individuals/family) or simply the fact that these traits do not possess dominance genetic variance (see *Discussion*). Including dominance variance in the models increased the AIC values for the models, indicating that GBLUP-AD models were overfitted compared to GBLUP-A models (Table 1), and that the simpler GBLUP-A models should be preferred.

The GBLUP-ADE analysis produced the most striking results with further reduction as to the additive genetic and the residual variances compared to the ABLUP and GBLUP-AD models for height and wood density, respectively (Table 1). This observed reduction in the additive genetic and residual variances was caused by the presence of significant additive × additive genetic variance within the total phenotypic variance (Table 1). This observed additive × additive genetic variance in turn resulted in further reduction of the narrow-sense heritability estimates; from 0.16 to 0.13 and from 0.30 to 0.18 in GBLUP-AD compared to GBLUP-ADE for height and wood density, respectively. Again, the GBLUP-ADE analysis did not cause any change to the dominance variances (Table 1). Small and not significant dominance × dominance and additive × dominance first-order interactions were observed for height and wood density in the GBLUP-ADE (Table 1). The AIC statistics for this model produced the best fit with values lower than that observed for all tested models for wood density (−9,726.65), supporting the inclusion of the additional epistasis terms in the model, specifically that of the additive × additive (Table 1). Unexpectedly, the AIC for GBLUP-A (17,465.80) produced the best fit for height (Table 1).

**Figure 2:**
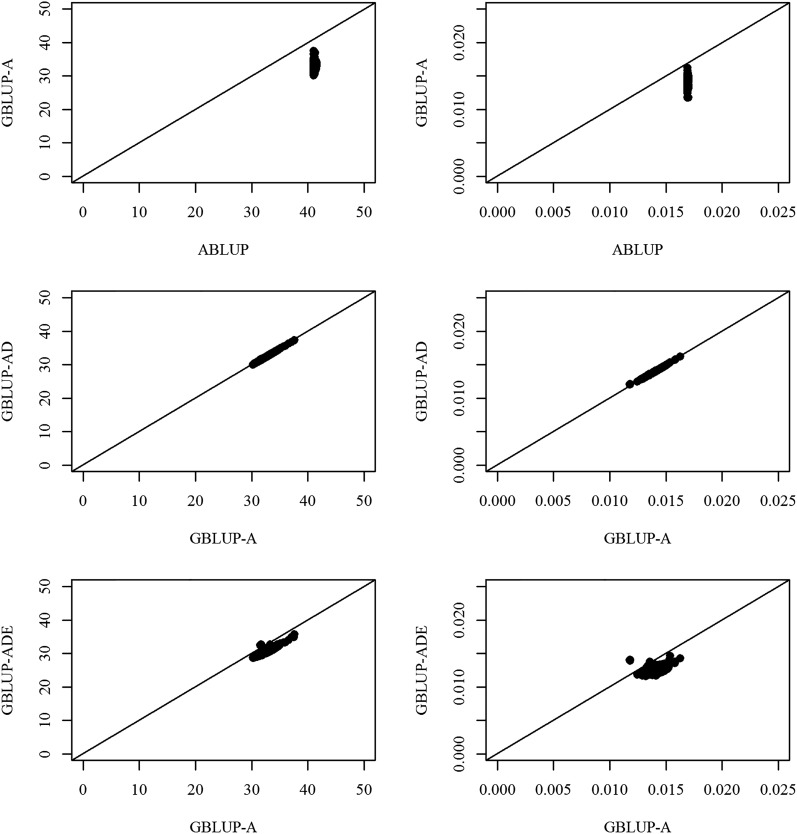
SEPs of BVs from the ABLUP (x-axis) against that from the GBLUP-A (y-axis) for height (left panel) and wood density (right panel) and that from the GBLUP-A against those from the GBLUP-AD and GBLUP-ADE.

**Figure 3:**
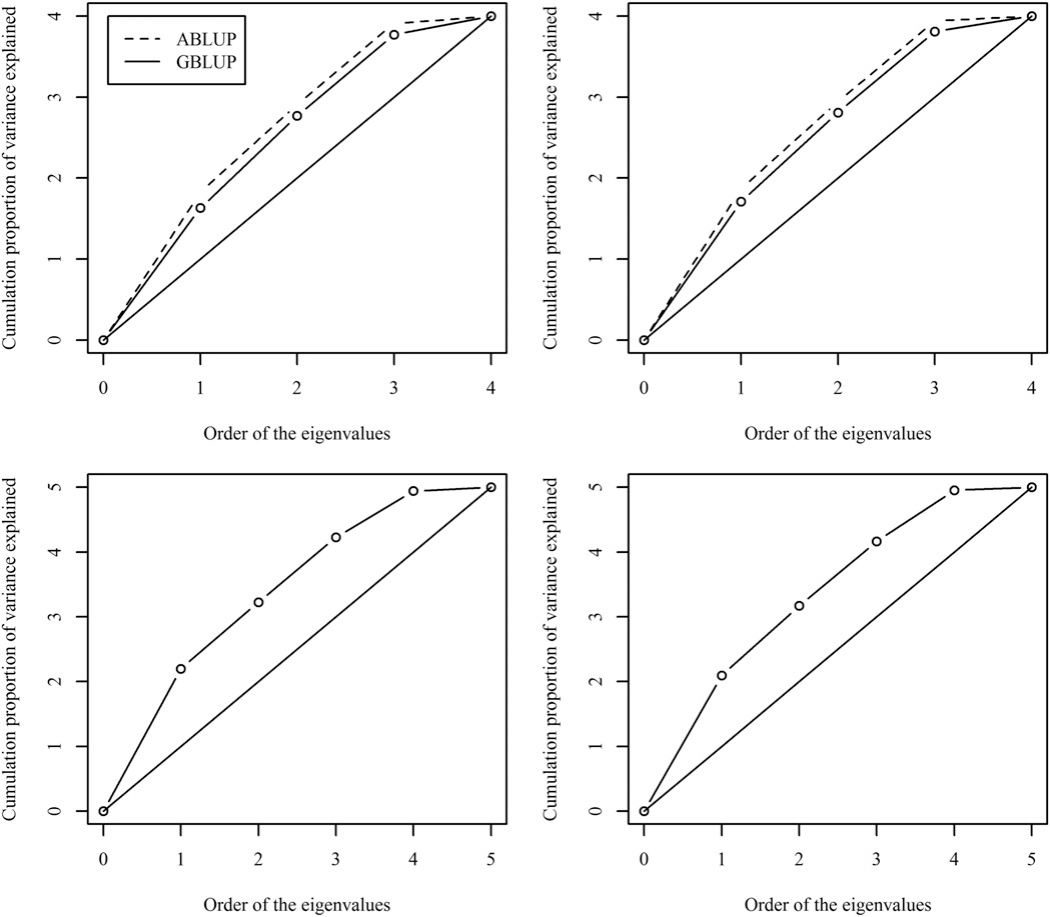
Cumulative proportion of the variance explained by eigenvalues for ABLUP *vs.* GBLUP-A (top panel) and GBLUP-ADE (bottom panel) for height (left) and wood density (right). Diagonal line represents an orthogonal correlation matrix.

Comparing the SEs for the predictions (SEPs) of breeding values (BV) between the ABLUP and GBLUP-A models, all of the SEPs for height and wood density BVs were smaller for GBLUP-A compared to ABLUP as all SEPs were below the 45° reference lines, clearly indicating the superiority of the GBLUP-A model (Figure 2). GBLUP-A and GBLUP-AD models produced identical results owing to the lack of significant dominance effects and all SEPs for height and wood density BVs resided on the diagonal 45° reference lines. Additionally, SEPs for height and wood density BVs from the GBLUP-ADE model were smaller than the corresponding SEPs produced by the GBLUP-A model indicating the effectiveness of the GBLUP-ADE model (Figure 2). When we compared the pedigree- and the marker-based models using the cumulative proportion of variance that was explained by eigenvalues of the sampling variance–covariance matrix of variance component estimates, we found that the GBLUP-A outperformed the pedigree-based (ABLUP) models as indicated by the closeness of their respective lines to the ideal scenario (straight line) where the variance components are completely independent (Figure 3). Finally, since the GBLUP-ADE model does not have a corresponding model in the pedigree method, GBLUP-ADE was plotted only against the 45° diagonal for reference (Figure 3).

Cross-validation prediction accuracies (Table 2; diagonals) indicated that the ABLUP model was associated with the lowest values among all tested models for both random and block restricted folding (range: 0.451−0.475 and 0.439−0.449 for height and wood density, respectively), while the GBLUP models produced greater prediction accuracies under the same two folding scenarios (range: 0.735−0.772 and 0.748−0.783, for height and wood density, respectively). Prediction accuracies were lowest under the family restricted scenario for the GBLUP models (range: 0.683−0.698 and 0.651−0.658, for height and wood density, respectively), with random folding producing the greatest prediction accuracies. Comparison of prediction accuracies among the GBLUP models using random folding showed that differences between GBLUP-A and GBLUP-AD were not significant (based on SEs); however the two were significantly greater than GBLUP-ADE for both height and wood density. The family and random folding scenarios both produced no significant differences in prediction accuracy among the GBLUP models.

**Table 2:** Correlations for height (HT) and wood density (WD) between estimated individual additive breeding values (EBVs) and predicted individual additive breeding values (PBVs) produced by 10-fold cross-validation for the four models (ABLUP, GBLUP-A, GBLUP-AD, and GBLUP-ADE) using random, block, and family based folding

	EBV – Full data							
	HT				WD			
PBV – Cross-Validation	ABLUP	GBLUP-A	GBLUP-AD	GBLUP-ADE	ABLUP	GBLUP-A	GBLUP-AD	GBLUP-ADE
Random folding								
ABLUP	**0.475 (0.003)**	0.407 (0.003)	0.407 (0.003)	0.401 (0.003)	**0.449 (0.004)**	0.554 (0.004)	0.554 (0.004)	0.523 (0.004)
GBLUP-A	0.331 (0.004)	**0.771 (0.003)**	0.770 (0.003)	0.772 (0.003)	0.402 (0.002)	**0.781 (0.001)**	0.781 (0.001)	0.773 (0.001)
GBLUP-AD	0.334 (0.003)	0.773 (0.002)	**0.772 (0.002)**	0.774 (0.002)	0.405 (0.004)	0.783 (0.003)	**0.783 (0.003)**	0.775 (0.003)
GBLUP-ADE	0.322 (0.004)	0.762 (0.003)	0.761 (0.003)	**0.765 (0.003)**	0.385 (0.002)	0.765 (0.002)	0.765 (0.002)	**0.773 (0.002)**
Block folding								
ABLUP	**0.451 (0.001)**	0.381 (0.001)	0.381 (0.001)	0.374 (0.001)	**0.439 (0.000)**	0.549 (0.000)	0.549 (0.000)	0.518 (0.000)
GBLUP-A	0.329 (0.000)	**0.735 (0.001)**	0.735 (0.001)	0.736 (0.001)	0.383 (0.000)	**0.748 (0.000)**	0.748 (0.000)	0.739 (0.000)
GBLUP-AD	0.328 (0.001)	0.734 (0.001)	**0.735 (0.001)**	0.736 (0.001)	0.383 (0.000)	0.748 (0.001)	**0.748 (0.000)**	0.740 (0.000)
GBLUP-ADE	0.313 (0.001)	0.711 (0.001)	0.712 (0.001)	**0.715** (0.001)	0.366 (0.000)	0.728 (0.001)	0.728 (0.001)	**0.733** (0.001)
Family folding								
ABLUP	NA*a*	NA	NA	NA	NA	NA	NA	NA
GBLUP-A	0.178 (0.011)	**0.683 (0.010)**	0.682 (0.010)	0.691 (0.009)	0.249 (0.006)	**0.651 (0.005)**	0.651 (0.005)	0.663 (0.005)
GBLUP-AD	0.188 (0.005)	0.692 (0.005)	**0.691 (0.005)**	0.699 (0.005)	0.254 (0.003)	0.656 (0.002)	**0.656 (0.002)**	0.668 (0.002)
GBLUP-ADE	0.190 (0.006)	0.691 (0.006)	0.689 (0.006)	**0.698 (0.006)**	0.228 (0.007)	0.627 (0.007)	0.627 (0.007)	**0.658 (0.006)**

Prediction accuracies are represented by bold diagonals and pairwise model correlations on the off-diagonals (SEs in parentheses).

a NA; predicted individual additive breeding value is equal to the overall mean of the model.

Pairwise model comparisons (Table 2; off-diagonals) showed high consistency between all GBLUP models within the individual folding scenarios. It is also noteworthy to mention that under the family folding scenario, the ability of ABLUP to produce across family prediction challenges the assumption of zero expected relatedness among OP families, thus predictions of individual additive BVs here would simply be equal to the overall mean.

## Discussion

Traditionally, the pedigree-based average numerator relationship matrix (***A***-matrix) is used to estimate the genetic variance components for forest tree progeny test populations. The estimated genetic variance components (*e.g.*, additive and dominance genetic variances, *etc*.) often are mating design-dependent and the mating scheme determines which component can be obtained. In most cases, this approach is incapable of disentangling the within-family genetic from within-family coenvironment effects. This is even more problematic in OP family screening as separating additive from nonadditive genetic variances is limited by shallow pedigrees and lack of connectedness among the tested families; furthermore, as shown in Table 1, the estimated additive genetic variance is inflated as the half-sib assumption is hardly fulfilled (Namkoong 1966; Squillace 1974; Askew and El-Kassaby 1994). In fact, the estimated genomic pairwise relationships of the 214 OP families studied showed deviation from the expected 0.25 coefficient of relatedness for half-siblings, confirming causes for additive genetic variance overestimation, while the relationships among members of unrelated families clustered around the expected 0.0 (Figure 1). The availability of dense genomic marker panels made it possible to genotype individuals for a large number of SNPs and obtain the realized genomic relationship matrix (***G***-matrix) among these individuals. In turn, the ***G***-matrix can be used as a substitute to the ***A***-matrices to estimate more accurate and precise genetic variance components as the ***G***-matrix represents the realized pedigree as well as having the capacity to exploit the Mendelian sampling/segregation within families (VanRaden 2008; Hayes *et al.* 2009). It is worthwhile to note that some of the recently reported gain increase in animal breeding programs is mainly due to exploiting the Mendelian sampling term (Avendano *et al.* 2004).

The utility of the ***G***-matrix in generating improved estimates of the genetic variance parameters from experimental populations of forest trees (*e.g.*, full-sib families) has recently been explored (Zapata-Valenzuela *et al.* 2013; Klápště *et al.* 2014; Muñoz *et al.* 2014). The present study, to our knowledge, represents the first attempt to implement the ***G***-matrix in OP family testing, thus not only overcoming the common bias associated with the unfulfilled half-sib assumption, but also separating the additive from the nonadditive genetic variance components. It is well known that separating the additive from the nonadditive (dominance and epistatic variances) genetic components requires elaborate mating designs with large numbers of interconnected full-sib families coupled with the inclusion of replicated clonal material (Foster and Shaw 1988; Bradshaw and Foster 1992; Costa *et al.* 2009). Our study accomplished a mixed-model approach for variance decomposition, providing realized estimates of the additive, dominance, and epistatic genetic variances without the need for mating designs to generate interconnected full-sib families or vegetative propagation for the production of replicated clonal material.

**Figure 4:**
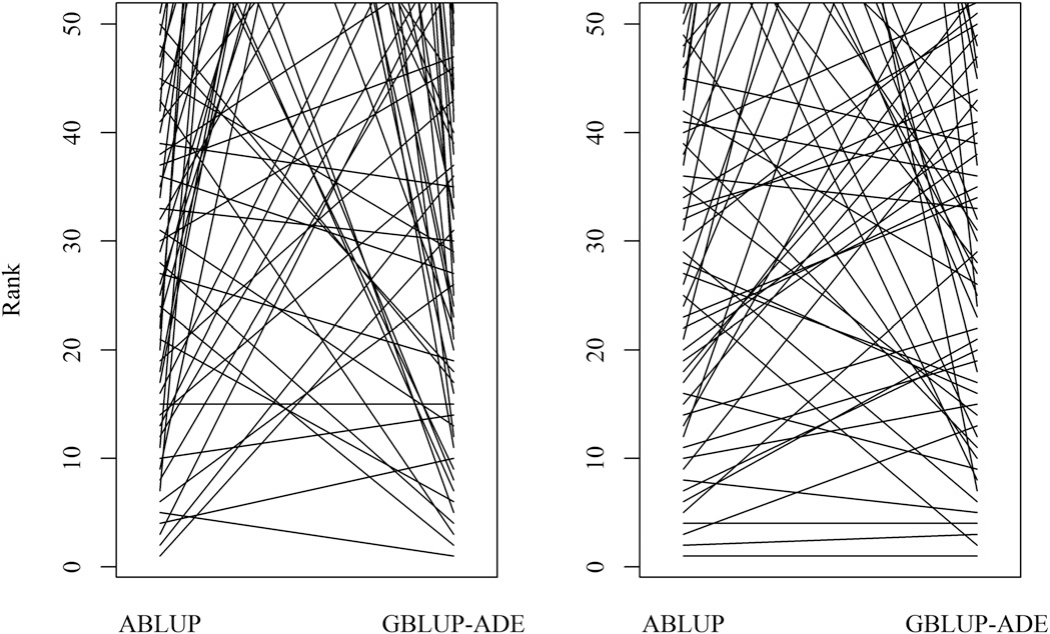
Ranking plots for the top 50 performing white spruce individuals for height (left) and wood density (right), respectively, comparing results of ABLUP *vs.* GBLUP-ADE assessments (note the number of highly ranked individuals in the ABLUP that dropped from the top 50 in the GBLUP-ADE).

It is interesting to note that the estimated additive genetic variances for the three realized genomic relationship matrix-based analyses (GBLUP-A, GBLUP-AD, and GBLUP-ADE) were lower than those of the average numerator relationship matrix (ABLUP) (Table 1), an observation already reported for mice (Lee *et al.* 2010), loblolly pine (Muñoz *et al.* 2014), and Brown Swiss cattle populations (Loberg *et al.* 2015). The improved performance of the GBLUP-A compared to that of the ABLUP indicates that the former model took full advantage of: (1) the within-family variation (*i.e.*, Mendelian sampling term), (2) discerning whether full-sibs, self-sibs, and self-halfs existed within the studied 214 OP families, (3) the ability to estimate among-family relationships (even if they were as small as those seen in Figure 1 and Figure 4) identifying pedigree errors if present, as shown in Figure 1 (*i.e.*, some individuals have a coefficient of relationship of 0.0 within the same OP family). The observed reduction in the additive genetic variance between the two models (ABLUP *vs.* GBLUP-A) resulted in concomitant increase in the residual error terms and hence considerably reduced narrow-sense heritability estimates for height (0.25 *vs.* 0.16) and also for wood density (0.61 *vs.* 0.30) along with improvement in the model fit based on improved AIC values (Table 1). Additionally, GBLUP-A produced greater precision for its estimated breeding value (EBV) as indicated by the EBV’s smaller SEs compared to the ABLUP (Figure 2; ABLUP *vs.* GBLUP-A).

It is noteworthy to mention that the present study is based on data collected from one site, thus there is a chance that the estimated genetic parameters could be upwards biased due to the genotype × environment confounding effects specific to this particular site or year. However, results from similar analyses for the same species and attributes for a set of 25 OP families planted in replicated trials over three sites in British Columbia were consistent with those reported here with the added benefit of estimating the additive and dominance × site (environment) interactions (O. Gamal El-Dien, unpublished data).

The observed overall trend in genetic variance decomposition persisted when the dominance genetic variance was estimated using the alternative genotypic approach proposed by Su *et al.* (2012) and discussed by Vitezica *et al.* (2013); however, the dominance genetic variance of wood density showed a slight increase (Supporting Information, Table S1).

Additionally, estimating the dominance genetic variance is only feasible when full-sib families are available (Zapata-Valenzuela *et al.* 2013; Klápště *et al.* 2014; Muñoz *et al.* 2014). This scenario is easily resolved when pedigree- and marker-based models are compared for mating design accommodating full-sib families (Muñoz *et al.* 2014). However, the utility of the GBLUP-AD model in OP family testing is still worth exploring to discern the dominance genetic variance – if existing – as well as separating the genetic variances from the confounding environment effects. It should be noted that the goodness-of-fit statistics (AIC) for the GBLUP-AD clearly indicated that adding the dominance genetic variance resulted in model over fit and this is expected due to the extremely small and nonsignificant dominance genetic variance (1.1 and 2.8% for height and wood density, respectively).

The model that included the additive, dominance, and epistatic variances (GBLUP-ADE) offered better partitioning of the variance complements, as the additive × additive epistatic variance became extremely pronounced and accounted for 11 and 52% of the total variance for height and wood density, respectively (Table 1 and Table S1). When we removed the dominance genetic variance from the GBLUP-ADE model, the revised models (GBLUP-AE) produced better model fit for height (17,466.9 *vs.* 14,472.9) and wood density (−9,732.6 *vs.* −9,726.6), confirming that dominance variance was negligible (Table 1). Interestingly, both models (GBLUP-ADE and GBLUP-AE) produced similar variance components apportionment and heritability estimates (Table 1, Figure S1, Figure S2, and Figure S3). Similar magnitude of the additive × additive epistatic variance to that of the additive variance, *per se*, was also observed in loblolly pine (Muñoz *et al.* 2014), a situation meeting theoretical expectations where the additive × additive epistatic variance is commonly absorbed by both the additive and the residual variances (Lynch and Walsh 1998; Jannink 2007; Mackay 2014). The power of the GBLUP-ADE and/or GBLUP-AE models in identifying and separating the additive × additive epistasis from the additive genetic variance lies in the genetic background of the tested families for providing a range of options to demonstrate all established interactions between the alleles at the various loci that are affecting the studied traits. The magnitude of the epistatic additive × additive genetic variance observed for height and wood density along with the AIC values produced from the tested models require some reflection. The observed AIC values support GBLUP-A and GBLUP-AE to be the best model for height and wood density, respectively (Table 1). However, in wood density where the additive × additive is ≈3 times that of the additive variance, the prediction accuracy of the GBLUP-AE and GBLUP-A models were almost similar (Table 1 and Table 2). This indicates that the additive × additive and additive relationship matrices are in a “tug-of-war” state over the same variance. In fact, we estimated the correlation between these two relationship matrices and it was close to perfect correlation (r = 0.988), confirming our notion and makes us believe that while we observed exceedingly large epistatic additive × additive genetic variance, the impact on predicting the BVs between GBLUP-A and GBLUP-AE is similar (Figure S4).

The subject of genetic epistasis is controversial as all variance components, including epistasis, are dependent on the allele frequencies in the studied population. Thus, epistasis could have an allusive and unique effect across different scenarios (Hill *et al.* 2008; Mackay 2014). The role of epistasis on the genetic architecture of quantitative traits is still not clearly determined due to several discrepancies between statistical and functional definition of epistasis. The statistical approach considers the epistatic variance orthogonal to the additive genetic variance and assumes a clear determination (separation) of both components by the implementation of independent terms in the model. Moreover, the epistatic effects are transient and disappear by breaking of linkage disequilibrium (LD) (Hill *et al.* 2008; Crow 2008, 2010). The functional approach assumes that the allelic substitution effect depends on the genetic background. Hill *et al.* (2008) based their empirical evidence on an exhaustive review across a wide range of species. This includes comparisons between narrow- and broad-sense heritability estimates, concluding that complex traits are mainly controlled by additive genetic variance as most studied cases supported the notion that the majority of the genetic variance appeared to be additive (Crow 2010). However, in the present study, if we utilized the heritability estimates derived from the GBLUP-A or GBLUP-AD models alone (without the application of the GBLUP-ADE and/or GBLUP-AE models), then the part of the genetic variance attributable to additive × additive interaction would have been excluded from the calculations, and thus our conclusion would have been mainly based on inflated additive genetic variance. Clearly, the utilization of the marker-based relationship method enabled disentangling the additive from the nonadditive genetic component, while effectively accounting for the proper environment variance through the removal of possible confounding effects. Such methodology provides much more realistic breeding value estimations for an individual.

Habier *et al.* (2007, 2010, 2013) indicated that the realized genomic relationships do not only capture relatedness among individuals but also the LD between SNPs and QTL, the deviation from independent segregation of alleles on the same gamete if the loci are linked (cosegregation or classical linkage), as well as the additive genetic relationship. Habier *et al.* (2013) demonstrated that these types of information collectively have different effects on the accuracy of the EBV. As a result, it is safe to state that there is more to the realized genomic relationship than the straightforward accounting for the Mendelian sampling term, hence resulting in the superior decomposition of the genetic variance components and BV estimation.

When ABLUP is used to estimate the genetic variance components from either half- or full-sib families, the above factors are barely considered, except those that were captured through common ancestry. As indicted above, the OP/half-sib structure is incapable of estimating the dominance and the epistatic genetic variances. This situation was clearly demonstrated in the study by Muñoz *et al.* (2014), as more accurate breeding value estimates and effective partitioning of variance components were obtained from their single site, full-sib, and clonally replicated loblolly pine experiment. The present study, on the other hand, demonstrated the power of the realized genomic relationship in quantitative genetic analyses using a more challenging structure (OP families). As proof-of-concept we compared the rank order among the top 50 performing individuals based on the conventional ABLUP *vs.* the GBLUP-ADE/GBLUP-AE (see details in the interaction plots of Figure 4). Only 23 and 33 of the top 50 individuals persisted from ABLUP to GBLUP-ADE/GBLUP-AE for height and wood density, respectively, and overall, the individuals’ ranking among the top 50 trees dramatically changed from ABLUP to GBLUP-AE. Interestingly, for the 10 best performing trees, only 2 and 4 individuals persisted for height and wood density, respectively (Figure 4). The true EBV of an individual is commonly determined from experiments with deep pedigree with ample connectedness; however, when the ABLUP approach is used in forestry progeny testing experiments that are characterized by shallow and inadequate connectedness, then the obtained breeding value is expected to greatly deviate from its true value as the assumption of mixed models of error-free covariance matrices is not met (Mrode 2014). The greatest difference between the GBLUP and ABLUP models is the ability of the former to more precisely define the genetic relationship between any two individuals as compared to the latter (Figure 1). Our model’s cross-validation supports this notion as the prediction accuracy for the GBLUP models was greater than those produced by ABLUP, regardless of the folding scenario (Table 2; diagonals). This difference is due to the quantity of realized pairwise genetic relationship information used for prediction, where in ABLUP only the information from the pedigreed OP family is used to predict the breeding value. Conversely in GBLUP all information from related individuals are used regardless of family assignment; this can be seen in the family folding scenario (Table 2). Thus, we believe that the EBVs produced by the GBLUP models are closer to the true value as more information is used.

We feel that our results have important and immediate implications for tree improvement programs in forestry as most programs are long-term and resource-dependent (El-Kassaby 1993). The conventional genetic improvement in forestry follows the classical recurrent selection scheme with repeated cycles of selection, breeding, and testing over time and space (Gamal El-Dien *et al.* 2015; Ratcliffe *et al.* 2015). These programs often include: (1) phenotypic selection of untested candidate parents from natural or managed forests, (2) propagation of the selected parents as grafts followed by a period of inactivity until sexual maturity, (3) the sexual production of structure pedigreed offspring from the selected parents using a specific mating design, (4) field testing over vast geographical areas for a reasonable period to attain meaningful data for target traits, (5) estimation of genetic parameters and ranking of individuals based on their BVs, and (6) genotypic selection of superior individuals for the second round of breeding and/or seed production from seed orchard populations. Obviously, the completion of a single breeding–testing–selection cycle is a protracted endeavor due to several uncontrollable biological factors; namely, the time needed for reaching sexual maturity for structured pedigree production and reproductive phenology and fertility variation that hinder the mating design completion (El-Kassaby *et al.* 1984, 1989; El-Kassaby and Barclay 1992). Therefore, the use of OP family testing, as demonstrated in the present study, allows immediate testing and the evaluation of a large number of individuals using their naturally produced offspring through wind pollination without the need for structured pedigree. The present study also demonstrated the utility of the realized genomic relationship approach in providing a simple and extremely efficient method for generating accurate genetic parameters from a simple OP testing that is characterized by shallow genealogy that is typical of most forest tree testing populations. It is noteworthy to mention that the use of the realized genomic relationship also allowed the generation of genetic parameters comparable to those generated only from elaborate mating designs coupled with cloning approaches. In conclusion, the utility of the realized genomic relationship in a simple, yet extremely efficient testing method, such as OP families, cannot be overlooked and calls for the re-evaluation of present-day conventional elaborate testing methods that are incapable of providing the genetic information produced in the present study.

## Supplementary Material

Supporting Information
