# Supplementary material for: Implementation of the Realized Genomic Relationship Matrix to Open-Pollinated White Spruce Family Testing for Disentangling Additive from Nonadditive Genetic Effects
Source: G3 (Bethesda). 2016 Jan 19;6(3):743–53. doi: 10.1534/g3.115.025957 (PMC4777135; doi:10.1534/g3.115.025957)
Supplement: Supporting Information [file supp_6_3_743__index.html]

Implementation of the Realized Genomic Relationship Matrix to Open-Pollinated White Spruce Family Testing for Disentangling Additive from Nonadditive Genetic Effects — Supporting Information 

# Implementation of the Realized Genomic Relationship Matrix to Open-Pollinated White Spruce Family Testing for Disentangling Additive from Nonadditive Genetic Effects

## Supporting Information for Gamal El-Dien *et al.*, 2016

**Files in this Data Supplement:**

- Figure S1 - Standard error of the predictions (SEP) of breeding values (BV) from the ABLUP (X-axis) against that from the GBLUP-A (y-axis) for height (left panel) and wood density (right panel) and that from the GBLUP-A against those from the GBLUP-AE. (.pdf, 133 KB)
- Figure S2 - Cumulative proportion of the variance explained by eigenvalues for ABLUP vs. GBLUP-A (left panel) and GBLUP-AE (right panel) for height (top) and wood density (bottom). Diagonal line represents an orthogonal correlation matrix. (.pdf, 74 KB)
- Figure S3 - Ranking plots for the top 50 performing white spruce individuals for height (left) and wood density (right), respectively, comparing results of ABLUP versus GBLUP-AE assessments (note; the number of highly ranked individuals in the ABLUP that dropped from the top 50 in the GBLUP-AE). (.pdf, 195 KB)
- Figure S4 - Ranking plots for the top 50 performing white spruce individuals for height (left) and wood density (right), respectively, comparing results of GBLUP-A versus GBLUP-AE assessments (note; the minor change in rank). (.pdf, 161 KB)
- Table S1 - Estimates of genetic variance components and their standard errors for height (HT) and wood density (WD) for the Québec white spruce population across the four genetic models utilizing the dominance matrix estimated through alternative genotypic approach proposed by Su *et al*. (2012) and discussed by Vitezica *et al*. 2013 (Genetics 195: 1223-1230). (.pdf, 208 KB)
