# Supplementary material for: Implementation of the Realized Genomic Relationship Matrix to Open-Pollinated White Spruce Family Testing for Disentangling Additive from Nonadditive Genetic Effects
Source: G3 (Bethesda). 2016 Jan 19;6(3):743–53. doi: 10.1534/g3.115.025957 (PMC4777135; doi:10.1534/g3.115.025957)
Supplement: Supporting Information [file supp_g3.115.025957_FigureS1.pdf]

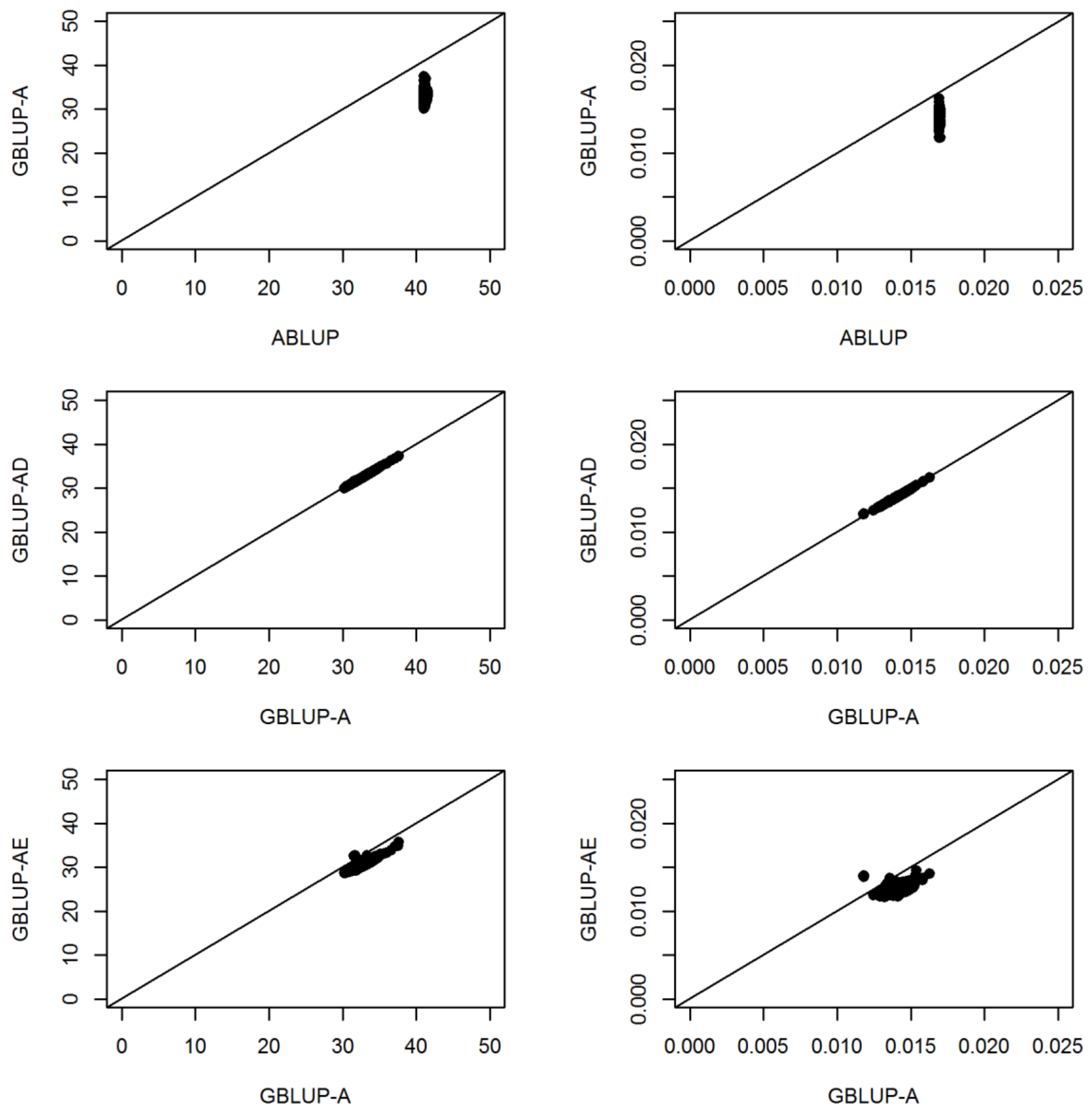

Figure S1: Standard error of the predictions (SEP) of breeding values (BV) from the ABLUP (X-axis) against that from the GBLUP-A (y-axis) for height (left panel) and wood density (right panel) and that from the GBLUP-A against those from the GBLUP-AE.
