# Supplementary material for: Implementation of the Realized Genomic Relationship Matrix to Open-Pollinated White Spruce Family Testing for Disentangling Additive from Nonadditive Genetic Effects
Source: G3 (Bethesda). 2016 Jan 19;6(3):743–53. doi: 10.1534/g3.115.025957 (PMC4777135; doi:10.1534/g3.115.025957)
Supplement: Supporting Information [file supp_g3.115.025957_FigureS2.pdf]

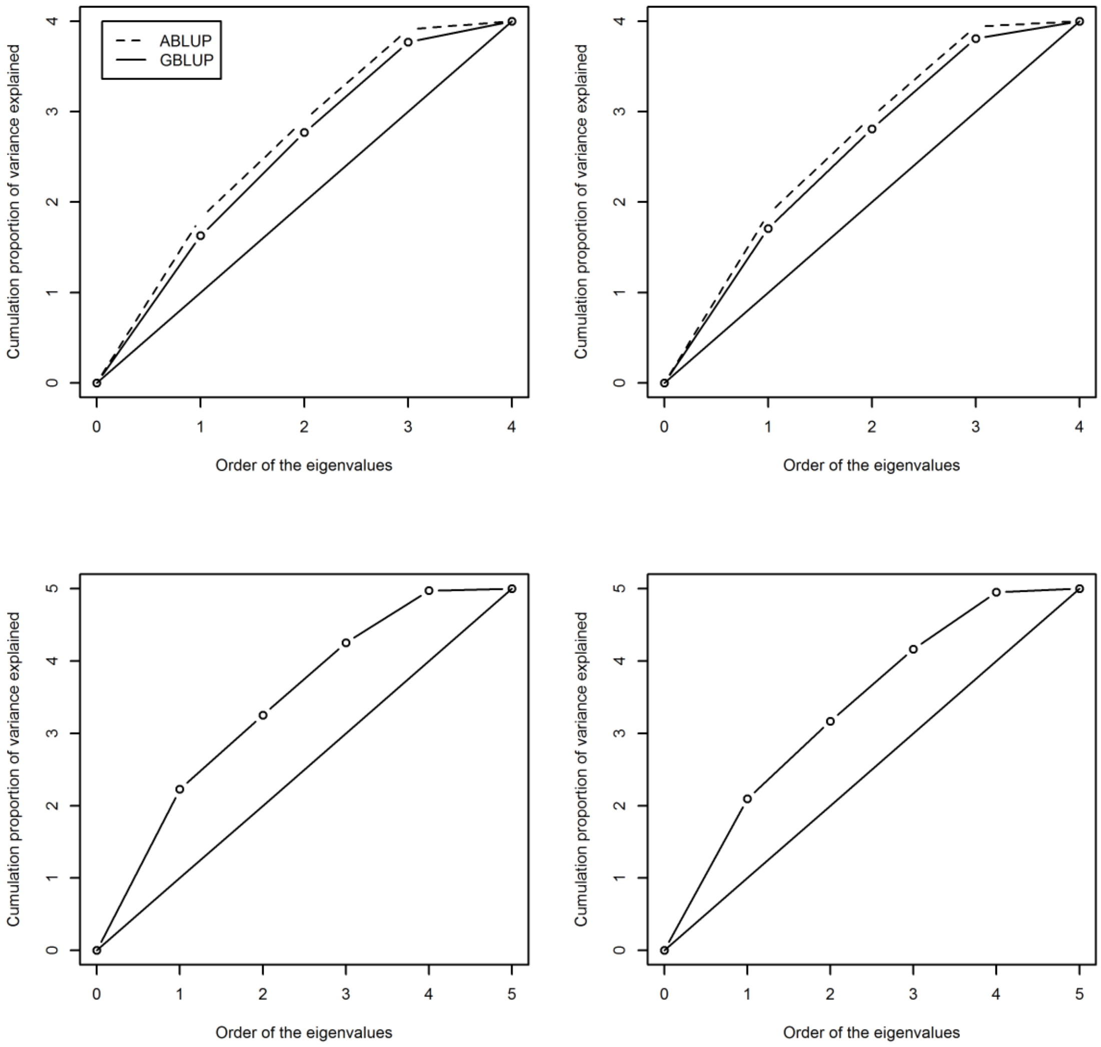

Figure S2: Cumulative proportion of the variance explained by eigenvalues for ABLUP vs. GBLUP-A (left panel) and GBLUP-AE (right panel) for height (top) and wood density (bottom). Diagonal line represents an orthogonal correlation matrix.
