# Supplementary material for: Implementation of the Realized Genomic Relationship Matrix to Open-Pollinated White Spruce Family Testing for Disentangling Additive from Nonadditive Genetic Effects
Source: G3 (Bethesda). 2016 Jan 19;6(3):743–53. doi: 10.1534/g3.115.025957 (PMC4777135; doi:10.1534/g3.115.025957)
Supplement: Supporting Information [file supp_g3.115.025957_FigureS4.pdf]

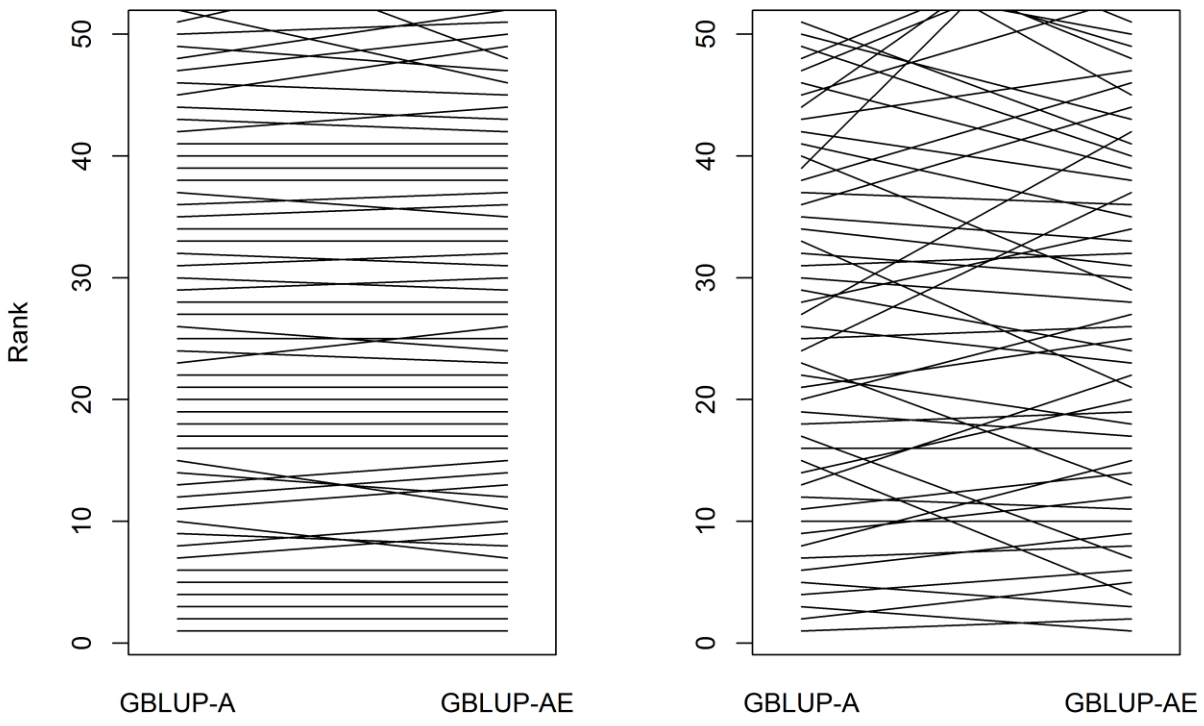

Figure S4: Ranking plots for the top 50 performing white spruce individuals for height (left) and wood density (right), respectively, comparing results of GBLUP-A versus GBLUP-AE assessments (note; the minor change in rank).
