# Supplementary material for: Implementation of the Realized Genomic Relationship Matrix to Open-Pollinated White Spruce Family Testing for Disentangling Additive from Nonadditive Genetic Effects
Source: G3 (Bethesda). 2016 Jan 19;6(3):743–53. doi: 10.1534/g3.115.025957 (PMC4777135; doi:10.1534/g3.115.025957)
Supplement: Supporting Information [file supp_g3.115.025957_TableS1.pdf]

Table S1. Estimates of genetic variance components and their standard errors for height (HT) and wood density (WD) for the Québec white spruce population across the four genetic models utilizing the dominance matrix estimated through alternative genotypic approach proposed by Su *et al.* (2012) and discussed by Vitezica *et al.* 2013 (Genetics 195: 1223-1230).

		**ABLUP**		**GBLUP-A**		**GBLUP-AD**		**GBLUP-ADE**		**GBLUP-AE**	
**Trait**	**S.O.V.**	**Value (SE)**	**%**	**Value (SE)**	**%**	**Value (SE)**	**%**	**Value (SE)**	**%**	**Value (SE)**	**%**
**HT**	$\boldsymbol{\sigma^2_{Rep}}$	561.40 (383.72)	4.70	554.81 (379.47)	4.68	558.75 (381.80)	4.71	557.67 (381.19)	4.71	555.15 (379.81)	4.69
	$\boldsymbol{\sigma^2_{F*Rep}}$	2,624.8 (497.90)	21.97	2,653.7 (479.62)	22.38	2,663.2 (479.19)	22.46	2,625.2 (481.03)	22.16	2613.1 (480.94)	22.07
	$\boldsymbol{\sigma^2_{A}}$	2,178.9 (879.65)	18.24	1,404.0 (413.19)	11.84	1,259.6 (457.28)	10.62	1,110.2 (500.29)	9.37	1,159.0 (480.98)	9.79
	$\boldsymbol{\sigma^2_{D}}$	N/A		N/A		350.24 (535.22)	**2.95**	215.63 (552.36)	**1.82**	N/A	
	$\boldsymbol{\sigma^2_{AA}}$	N/A		N/A		N/A		1134 (1653.9)	9.57	1,352.7 (1595.6)	11.43
	$\boldsymbol{\sigma^2_{DD}}$	N/A		N/A		N/A		7.79E-03 (1.75E-03)	0.00	N/A	
	$\boldsymbol{\sigma^2_{AD}}$	N/A		N/A		N/A		5.09E-03 (1.14E-03)	0.00	N/A	
	$\boldsymbol{\sigma^2_{E}}$	6,581.7 (808.23)	55.09	7,243.6 (535.33)	61.10	7,028.6 (622.61)	59.26	6,203.8 (1391.6)	52.37	6,159.1 (1390.9)	52.02
	$\boldsymbol{h^2}$	0.249 (0.095)		0.162 (0.046)		0.146 (0.051)		0.128 (0.057)		0.134 (0.054)	
	**AIC**	17,478.64		17,465.80		17,467.30		17,472.76		17,466.94	
**WD**[1]	$\boldsymbol{\sigma^2_{Rep}}$	1.36E-05 (1.11E05)	1.07	1.24E-05 (1.04E-05)	1.01	1.24E-05 (1.04E-05)	1.01	1.32E-05 (1.09E-05)	1.09	1.34E-05 (1.10E-05)	1.10
	$\boldsymbol{\sigma^2_{F*Rep}}$	2.47E-05 (4.77E-05)	1.95	5.89E-05 (4.70E-05)	4.78	5.39E-05 (4.66E-05)	4.37	4.38E-05 (4.63E-05)	3.61	4.65E-05 (4.65E-05)	3.83
	$\boldsymbol{\sigma^2_{A}}$	7.48E-04 (1.28E-04)	59.01	3.51E-04 (5.52-E05)	28.50	2.91E-04 (5.93E-05)	23.63	1.78E-04 (6.07E-05)	14.65	2.07E-04 (5.85E-05)	17.05
	$\boldsymbol{\sigma^2_{D}}$	N/A		N/A		1.48E-04 (6.78E-05)	**11.99**	9.64E-05 (6.64E-05)	**7.94**	N/A	
	$\boldsymbol{\sigma^2_{AA}}$	N/A		N/A		N/A		5.71E-04 (1.37E-04)	46.98	6.32E-04 (1.34E-04)	52.03
	$\boldsymbol{\sigma^2_{DD}}$	N/A		N/A		N/A		1.54E-10 (5.36E-11)	0.00	N/A	
	$\boldsymbol{\sigma^2_{AD}}$	N/A		N/A		N/A		5.00E-10 (1.75E-10)	0.00	N/A	
	$\boldsymbol{\sigma^2_{E}}$	4.81E-04 (1.12E-03)	37.96	8.10E-04 (6.28E-05)	69.71	7.26E-04 (7.20E-05)	59.00	3.13E-04 (1.09E-04)	25.73	3.16E-04 (1.11E-04)	25.98
	$\boldsymbol{h^2}$	0.609 (0.093)		0.303 (0.043)		0.250 (0.048)		0.154 (0.051)		0.179 (0.049)	
	**AIC**	-9,687.42		-9,716.32		-9,719.42		-9,728.84		-9,732,64	

[1]log transformation
